# How to manage anaphylaxis in primary care

**DOI:** 10.1186/s13601-017-0182-7

**Published:** 2017-12-11

**Authors:** Alberto Alvarez-Perea, Luciana Kase Tanno, María L. Baeza

**Affiliations:** 1 Allergy Service, Hospital General Universitario Gregorio Marañón, Doctor Esquerdo, 46, 28007 Madrid, Spain; 2 Gregorio Marañón Health Research Institute, Madrid, Spain; 3 Hospital Sírio Libanês, São Paulo, Brazil; 4 Division of Allergy, Department of Pulmonology, University Hospital of Montpellier, Montpellier, France; 5 Pierre and Marie Curie Institute of Epidemiology and Public Health, Sorbonne Universités, Paris, France; 6 Biomedical Research Network on Rare Diseases (CIBERER)-U761, Madrid, Spain

**Keywords:** Anaphylaxis, Epinephrine, Management, Primary care

## Abstract

Anaphylaxis is defined as a severe life-threatening generalized or systemic hypersensitivity reaction characterized by rapidly developing airway and/or circulation problems. It presents with very different combinations of symptoms and apparently mild signs and can progress to fatal anaphylactic shock unpredictably. The difficulty in recognizing anaphylaxis is due, in part, to the variability of diagnostic criteria, which in turn leads to a delay in administration of appropriate treatment, thus increasing the risk of death. The use of validated clinical criteria can facilitate the diagnosis of anaphylaxis. Intramuscular epinephrine (adrenaline) is the medication of choice for the emergency treatment of anaphylaxis. Administration of corticosteroids and H1-antihistamines should not delay the administration of epinephrine, and the management of a patient with anaphylaxis should not end with the acute episode. Long-term management of anaphylaxis should include avoidance of triggers, following confirmation by an allergology study. Etiologic factors suspected in the emergency department often differ from the real causes of anaphylaxis. Evaluation of patients with a history of anaphylaxis should also include an assessment of personal data, such as age and comorbidities, which may increase the risk of severe reactions. Special attention should also be paid to co-factors, as these may easily confound the cause of the anaphylaxis. Patients experiencing anaphylaxis should administer epinephrine as soon as possible. Education (including the use of Internet and social media), written personalized emergency action plans, and self-injectable epinephrine have proven useful for the treatment of further anaphylaxis episodes.

## Background

Anaphylaxis is defined as a severe life-threatening generalized or systemic hypersensitivity reaction [1, 2]. All anaphylaxis guidelines [1–5] highlight the severity of the anaphylactic episode and the risk of death. Since anaphylaxis is characterized by rapidly developing life-threatening airway and/or circulation problems, it must be managed quickly. However, anaphylaxis is often difficult to recognize owing, in part, to the variability of diagnostic criteria, which in turn leads to a delay in administration of appropriate treatment, thus increasing the risk of death. In addition, it hampers reliable epidemiological data since medical records are the basis of national and international registries.

Primary care physicians have a pivotal role in the prevention and treatment of anaphylaxis. However, few studies have covered the management of anaphylaxis in primary care. A systematic review on the management of anaphylaxis identified a number of gaps at this level, most notably a lack of knowledge regarding recognition of the reaction, treatment with epinephrine (adrenaline), and prescription of epinephrine auto-injectors (EAI) [6]. The most common approach to the evaluation of the management of anaphylaxis in primary care has been through questionnaires and case studies. The results of several recent surveys from different countries are based on data from general practitioners, paramedics, and, most frequently, paediatricians and do not differ much from one study to another. There is still much room for improvement with respect to knowledge about epinephrine as the initial treatment of anaphylaxis, intramuscular administration, doses, and prescription of EAIs [7–12]. Studies that reviewed healthcare databases in Canada [13, 14] and The Netherlands [15] reported similar findings. Interdisciplinary communication and education on anaphylaxis are the most frequently proposed solutions.

Awareness of anaphylaxis as a life-threatening medical condition has been increasing in various specialties, and recent publications indicate that the condition is not as uncommon as previously perceived. Epidemiological data cite incidence rates ranging from 1.5 to 7.9/100,000 person-years in Europe [16] and 1.6 to 5.1/100,000 person-years in the United States [17]. However, epidemiological data on the morbidity and mortality of anaphylaxis are still not optimal. Most studies are biased, mainly because of their limited external validity. Variability in methodology, selection of specific populations, and the frequent use of cumulative incidence rates hamper the extrapolation of results to other populations.

To date, most population-based studies that document allergic reactions using the International Classification of Diseases (ICD) report inconsistent data [17–20], thus hampering determination of the prevalence and incidence of severe allergic reactions, such as anaphylaxis. However, studies have calculated the prevalence of anaphylaxis using different approaches such as emergency department (ED) records or number of EAIs prescribed. Studies on the incidence of anaphylaxis in the ED report rates ranging from 0.04 to 0.5% of visits [20–28]. This remarkable variability is related to differences between populations, characteristics of the ED, difficulties recognizing at-risk and anaphylactic patients, and methodology applied to record the rates. Data on mortality are sparse, and publications show considerable variability, ranging from 0.04 to 2.7 cases/million/year [29–31]. It has been estimated that 1 in every 3000 inpatients in American hospitals experience an anaphylactic reaction with a risk of death of around 1%, that is, 500–1000 deaths annually in the US [32]. Brazilian data suggest that the mortality rate of anaphylaxis is 1.1/million/year and that reactions are triggered mainly by drugs. In addition, deaths typically occurred in hospitals, including both the ED and patients who were dead on arrival [31].

Anaphylaxis typically occurs through an IgE-dependent immunologic mechanism and is most commonly triggered by foods, stinging insect venom, and medications, although pathophysiological events such as IgE-independent immunologic mechanisms and direct mast cell stimulation are also involved [2]. Several studies have demonstrated the complexity of mast and basophil cell signalling and the sensitivity of this system to regulation by specific pathways. A wide variety of molecules contribute to the activation of mast cells and the release of mediators (IgE, IgG, stem cell factor, complement proteins, cytokines, neuropeptides, and opioids), which may interact with receptors on the surface of mast cells, as summarized by Gurish and Castells [33]. Nevertheless, most of their mechanisms are not fully understood [34–37].

## Diagnosis of anaphylaxis

As anaphylaxis is a rapidly evolving condition affecting several systems, clinical diagnosis is based on consideration of the signs and symptoms that appear within 2 h of exposure to the allergen or trigger [38]. Rapid diagnosis ensures optimal management. The signs and symptoms include respiratory distress, hypotension, tachycardia, cyanosis, urticaria, angioedema, nausea, vomiting, diarrhoea, and abdominal pain. In general, cutaneous manifestations are observed in most cases, followed in frequency by cardiovascular and respiratory symptoms [39]. Diagnosis is more challenging when cutaneous symptoms are absent. Such is the case of hypotensive shock with no other symptoms in the context of contact with a known or suspected allergen. Respiratory (e.g., inspiratory difficulty, dysphonia, and sialorrhoea) and cardiovascular manifestations (e.g., sudden reduced blood pressure and tachycardia) are potentially life-threatening features of anaphylaxis and should be considered warning signs [1–5].

One of the key challenges in recognizing anaphylaxis is that the combination of signs and symptoms is not always the same and reactions with mild and moderate severity may not be easily recognized as anaphylaxis by physicians who are unfamiliar with the condition. Therefore, the use of validated clinical criteria can be helpful when diagnosing anaphylaxis. Previously published criteria (Table 1) have proven to be sufficiently sensitive and accurate for the diagnosis of anaphylaxis in the ED [40].

**Table 1:** Diagnostic criteria for anaphylaxis, adapted [1]

*Diagnostic criteria for anaphylaxis*
Anaphylaxis is highly likely when any *one* of the following three criteria is fulfilled
1. Acute onset of an illness (minutes to several hours) with involvement of the skin, mucosal tissue, or both (e.g., generalized hives, pruritus or flushing, swollen lips–tongue–uvula and at least one of the following
a. Respiratory compromise (e.g., dyspnea, wheeze–bronchospasm, stridor, reduced PEF, hypoxemia)
b. Reduced BP or associated symptoms of end-organ dysfunction (e.g., hypotonia [collapse], syncope, incontinence)
2. Two or more of the following that occur rapidly after exposure to a *likely* allergen for that patient (minutes to several hours)
a. Involvement of the skin–mucosal tissue (e.g., generalized hives, pruritus, flushing, swollen lips–tongue–uvula
b. Respiratory compromise (e.g., dyspnea, wheeze–bronchospasm, stridor, reduced PEF, hypoxemia)
c. Reduced BP or associated symptoms (e.g., hypotonia [collapse], syncope, incontinence)
d. Persistent gastrointestinal symptoms (e.g., crampy abdominal pain, vomiting)
3. Reduced BP after exposure to *known* allergen for that patient (minutes to several hours)
a. Infants and children: low systolic BP (age specific) or > 30% decrease in systolic BP^a^
b. Adults: systolic BP of < 90 mmHg or > 30% decrease from that person’s baseline

*PEF* peak expiratory flow, *BP* blood pressure

^a^Low systolic blood pressure for children is defined as < 70 mmHg from 1 month to 1 year, less than (70 mmHg + [2 × age]) from 1 to 10 years, and < 90 mmHg from 11 to 17 years

Over the last few decades, in vitro and in vivo methods have been developed and applied to support the clinical diagnosis of anaphylaxis and to reach the etiological diagnosis of the reaction [41].

Accurate clinical data in the ED, together with available in vitro tools, can ensure a correct diagnosis of anaphylaxis. The in vitro diagnosis of anaphylaxis includes serial measurement of the mediators released during an anaphylactic reaction, namely, tryptase, histamine, chymase, carboxypeptidase A3, platelet-activating factor, and other products from mastocytes. Measurement of serum (or plasma) tryptase levels is recommended in the diagnostic workup of systemic anaphylaxis, although the results should be interpreted on an individual basis and considering the complete allergy workup [41]. During anaphylaxis, serum tryptase peaks 60–90 min after the onset of the reaction and, in general, starts to decrease after 120 min. Therefore, for the diagnosis of anaphylaxis, blood samples should be collected within 1–2 h of the reaction and after 24 h in order to detect this decrease [42]. However, normal levels of serum tryptase in the first sample do not exclude anaphylaxis. Other biomarkers, such as histamine and its metabolites, chymase, carboxypeptidase, cysteinyl leukotrienes, prostaglandins, or platelet-activating factor, have lower and variable positive predictive values for a diagnosis of anaphylaxis than serum tryptase [42].

The identification of agents which trigger the anaphylactic reaction is essential for prevention of new exposure and recurrence. In general, diagnostic testing should be performed 3–4 weeks after the acute episode to allow time for the recovery of mast cell activity [43, 44]. The etiological diagnosis can be supported by serologic methods, e.g., allergen-specific serum IgE, with cellular tests, which measure the release of basophil mediators (leukotrienes, histamine), or with the basophil activation test, in which the expression of basophil markers is analyzed [41]. These techniques offer interesting alternatives in the diagnosis of potential triggers of anaphylaxis. The basophil activation test provides important advantages in patients with anaphylaxis to β-lactams, non-steroidal anti-inflammatory drugs, neuromuscular blocking agents, and drugs for which there is no technique to measure specific IgE [45]. Although in vitro tests are safer, their sensitivity and specificity remain to be determined.

The main in vivo tests currently used to investigate allergy and hypersensitivity reactions are skin tests and provocation tests [41], which follow standard methods and practice parameters and should be requested, performed, and interpreted by experienced professionals.

Co-factors, or augmenting factors, such as concomitant asthma, exercise, or specific drugs (e.g., non-steroidal anti-inflammatory drugs, ACE inhibitors) (Table 2), must always be considered. Co-factors may lead to more severe reactions or to anaphylaxis with lower doses of allergen. Physical exercise is one of the best-known augmenting factors in anaphylaxis. In fact, food-dependent exercise-induced anaphylaxis is considered a distinct clinical syndrome [46]. Sensitization to ω-5 gliadin most commonly presents as wheat-dependent exercise-induced anaphylaxis [47]. In general, the mechanisms underlying the role of co-factors in anaphylaxis remain poorly understood [48].

**Table 2:** Most common co-factors of anaphylaxis

Drugs
NSAIDs
ACE inhibitors
β-blockers
Alcohol
Physical exercise
Psychogenic stress
Hormonal cycle
Concomitant diseases
Asthma
Infections
Cardiovascular disease
Mastocytosis

*NSAID* non-steroidal anti-inflammatory drug, *ACE* angiotensin-converting enzyme

## Acute management of anaphylaxis

Anaphylaxis is a life-threatening medical emergency, and prompt evaluation and intervention are critical for its management. All health professionals should be prepared to identify and treat patients with anaphylaxis. An apparently mild presentation may unpredictably progress to fatal anaphylactic shock in minutes [49]. The severity of an anaphylactic episode can differ from one patient to another, and even in the same patient from one episode to another [50].

**Fig. 1:**
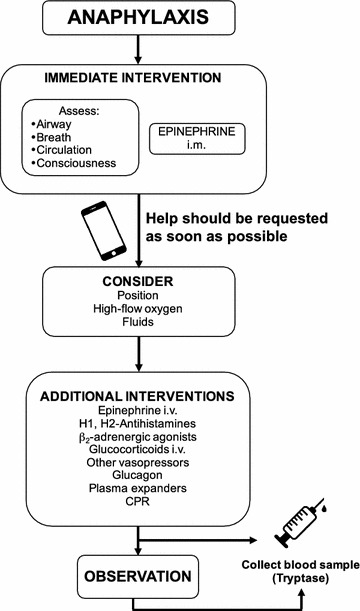
Algorithm for the acute management of anaphylaxis

The management of a patient with anaphylaxis should start with the removal of exposure to the known or suspected trigger, if still possible [51], followed by the assessment of patient’s circulation, airway patency, breathing, mental status, skin, and, if possible, weight [44] (Fig. 1).

After administration of epinephrine, patients with anaphylaxis should be placed supine with their lower limbs elevated. They should not be placed seated, standing, or in the upright position. In cases of vomiting or dyspnoea, the patient should be placed in a comfortable position with the lower limbs elevated. This should prevent distributive shock and empty vena cava/empty ventricle syndrome [52].

Help should be requested as soon as possible. Patients’ vital signs (blood pressure, heart frequency, and oxygenation) should be monitored continuously or as often as possible. When indicated, supplemental oxygen and intravenous fluid should be administered and, if necessary, cardiopulmonary resuscitation should be performed [53].

Biphasic anaphylaxis is defined as recurrence of anaphylaxis hours after recovery of the initial symptoms, with no further exposure to the trigger [1]. Given that biphasic anaphylaxis is not uncommon [21, 54], patients overcoming symptoms should undergo monitoring and medical supervision in a centre with trained staff, an ED, and hospital beds available. The duration of monitoring must be tailored to the severity of symptoms [55].

## Pharmacologic treatment of anaphylaxis: epinephrine as the drug of choice

Evidence supporting the use of different medications for the treatment of anaphylaxis is based on observational, epidemiologic, pharmacologic, and animal models, as well as on post-mortem studies [56]. The severity of anaphylaxis makes epinephrine difficult to assess in prospective, randomized, double-masked, placebo-controlled trials [57].

Epinephrine is the medication of choice for the immediate treatment of anaphylaxis [58] and is the only drug that exerts a vasoconstrictor effect, thus reverting airway mucosal edema and hypotension [59]. Additionally, it has inotropic and chronotropic cardiac effects, bronchodilator activity and a stabilization effect on mast cells and basophils [60, 61].

Evidence has shown that delayed injection of epinephrine is associated with higher hospitalization and mortality rates [62, 63]. In contrast, prompt pre-hospital administration of epinephrine is associated with better outcomes [64, 65].

Epinephrine should be injected by the intramuscular route in the *vastus lateralis* muscle (outer thigh) due to its vasodilator effect in skeletal muscle, which facilitates rapid absorption and pharmacologic effects. In contrast, it acts as a vasoconstrictor in the subcutaneous tissue, potentially delaying its absorption [66–68].

The dose of epinephrine for the treatment of anaphylaxis in a health centre is 0.01 mg/kg when administered intramuscularly at a 1:1000 dilution. The maximum dose is 0.3 mg for children and 0.5 for teenagers and adults. With an EAI, patients weighing between 7.5 and 25 kg should receive 0.15 mg, while patients weighing over 25 kg should receive 0.3 mg [3].

The epinephrine injection can be repeated once or twice at 5–15 min intervals in patients who do not respond to the first dose, in patients whose reaction is progressing rapidly, or in biphasic anaphylaxis [69].

A third dose of epinephrine is needed less frequently [70, 71]. Lack of response to epinephrine is an indicator of the need for admission to the intensive care unit, where the patient can receive further care, such as intravenous infusion of epinephrine [72].

Administration of therapeutic doses of epinephrine, as used in anaphylaxis, may induce adverse effects, including transient anxiety, headache, dizziness, tremor, pallor, and palpitations. These symptoms are similar to those caused physiologically by increased endogenous epinephrine levels. However, the adverse effects cannot be dissociated from the beneficial effects of epinephrine [57, 60, 61, 73]. Less frequently, usually due to overdosing or the administration of an intravenous bolus, epinephrine may cause ventricular arrhythmias, pulmonary oedema, malignant hypertension, and intracranial haemorrhage, although these effects are very rare in children and healthy adults [59, 61, 74, 75].

There is no absolute contraindication to epinephrine in the treatment of anaphylaxis [50]. However, the risk–benefit ratio should be assessed in patients with cardiovascular disease [76]. The heart is a potential target organ in anaphylaxis, and acute coronary syndrome can occur during anaphylaxis in the absence of epinephrine [77].

## Second-line drugs for the treatment of anaphylaxis

Antihistamines (both anti-H1 and anti-H2) and corticosteroids are second-line medications for the treatment of anaphylaxis, since they are not life-saving and, therefore, should not be used as initial or only treatment [58, 78, 79].

There is no evidence that supports the use of H1-antihistamines in anaphylaxis. H1-antihistamines relieve itching, flushing, and urticaria, but they do not act on airway obstruction or hypotension. Their onset of action is slower than that of epinephrine. Moreover, recommendations for anaphylaxis, including the doses administered, are extrapolated from those used in urticaria. A limited number of first-generation H1-antihistamines is available in parenteral form for use in anaphylaxis. These drugs frequently cause mild side effects (e.g., somnolence, confusion). Severe adverse effects (e.g., seizures, hypotension, cardiac toxic events) are uncommon. Second-generation H1-antihistamines are more secure; however, they are not available for parenteral use. Nevertheless, antihistamines are still the most frequently wrongly used drugs for the treatment of anaphylactic reactions in the ED [58, 80, 81].

There is evidence that the effect of H2-antihistamines, when administered concurrently with H1-antihistamines, could be enhanced in skin symptoms, although their role in anaphylaxis remains unclear [79, 82].

Corticosteroids are traditionally administered to prevent biphasic or protracted anaphylaxis, although these effects have never been proven. Their use in asthma indicates that the onset of pharmacological action may take several hours after administration. Therefore, corticosteroids have little or no effect on initial symptoms or signs [78].

Inhaled beta-2 adrenergic agonists, such as salbutamol or terbutaline, may play a role in anaphylaxis by relieving bronchospasm, in addition to the effect of epinephrine. However, the administration of these drugs should never delay the administration of epinephrine [2].

## Long-term management of anaphylaxis

Management of anaphylaxis continues after resolution of the acute episode. The key to preventing future anaphylactic reactions is a confirmed etiological diagnosis and the avoidance of triggers. In some cases, long-term etiologic treatments may provide protection in case of accidental exposures, such as allergen-specific immunotherapy in cases of *Hymenoptera* venom-induced anaphylaxis. Finally, the patient should know how to treat new symptoms in case they re-appear [2–5, 83].

All patients who experience an episode of anaphylaxis should be advised that their specific triggers must be identified. Important differences between the etiological diagnosis suspected in the ED and the definitive cause of anaphylaxis have been reported in recent studies in adults and children [28, 84, 85]. The triggers of anaphylaxis can be identified by allergy specialists, who will also provide information on possible cross-reacting agents and safe alternatives, especially in the case of drug hypersensitivity. Such an approach has proven useful for reducing the risk of severe anaphylaxis [86]. The tools most commonly used by allergists to this end are a detailed history/documentation of the acute episode, skin tests, detection of allergen-specific IgE, and challenge tests. It is usually accepted that the optimal time for testing is around 4 weeks after the acute episode [5].

Avoidance of some triggers may impact negatively on patients’ quality of life [50]. In these cases, immunomodulatory and/or etiological treatments may be available, including drug desensitization [87], insect venom immunotherapy [88], food oral immunotherapy [89], and anti-IgE therapy [90].

Given the unpredictable nature of anaphylaxis, patients should be prepared to act whenever necessary, especially when health care professionals are not present. International guidelines consider written action plans to be a useful tool for optimizing outcome [2–5].

An anaphylaxis action plan is a written document that can guide the patient and caregivers in the event that he or she experiences an allergic reaction in the community (Table 3). The several available action plan models have improved outcomes for other allergic diseases, such as asthma, and thus have the potential to reduce the frequency and severity of reactions, as well as the anxiety felt by patients and their caregivers [91].

**Table 3:** Summary of data that should be included in a personalized anaphylaxis emergency action plan

Patient identification (name, address, date of birth, weight)
Photograph
Specific allergens
Specific co-factors and risk factors
Instructions on when to use epinephrine, including dosage
Additional medications, including instructions and dosage
Details of contact person
Telephone number of the local emergency service
Physician (allergist, family doctor)

EAIs are the preferred method for administration of epinephrine in the community setting. Given that handling of ampoules, needles, and syringes by patients or their relatives is often subject to error, the EAI could be preferable when commercially available [2–5]. Currently, EAIs administer three doses, namely, 0.15, 0.3 mg, and, in a minority of countries, 0.5 mg. Self-injectable epinephrine may also be used in health care settings [92].

Self-injectable epinephrine should be prescribed to patients with a history of anaphylaxis and a high probability of recurrence, especially when triggered by foods or insects and in patients with idiopathic anaphylaxis. Patients living in isolated areas without access to medical services, and patients with mastocytosis, should also receive EAIs (Table 4) [2–5].

**Table 4:** Indications for prescription of epinephrine auto-injectors

Cases requiring *at least one* epinephrine autoinjector device	Cases requiring *more than one* autoinjector device
History of a previous anaphylactic reaction	High body weight
Allergy to ubiquitous triggers (peanut, egg, milk)	History of anaphylaxis requiring more than one dose of epinephrine
Clinical reactions even to tiny amounts of food, excluding oral allergy syndrome	History of protracted or biphasic anaphylaxis
Food allergy and unstable or moderate to severe asthma	Fear of possible misuse
Remote from medical help and previous mild to moderate reactions	Food allergy and severe asthma
Underlying mastocytosis	

Specific patients with no history of anaphylaxis should also keep an EAI at home. These cases include patients with previous generalized skin reactions after exposure to trace amounts of food and those who are allergic to triggers that are difficult to avoid owing to their ubiquity (e.g., peanut, egg, milk) (Table 4) [2–5].

The number of devices prescribed should be considered. General indications for prescribing 2 or more EAIs include high body weight, fear of possible misuse, a history of biphasic or protracted reactions in the past, and concomitant severe asthma (Table 4) [93].

Nevertheless, prescription of an EAI must be based on objective data from the medical history after the risk–benefit ratio has been properly assessed. Carrying an EAI has been associated with impaired quality of life [94].

There is growing evidence on the benefits of education with the aim of reducing the morbidity and mortality of anaphylaxis, although long-term benefits have yet to be clarified [95, 96]. Education should begin after the resolution of the acute episode, before discharge, and ED health professionals should be well prepared to provide correct guidance. Patients should be taught how to recognize anaphylaxis symptoms, when to inject epinephrine and seek medical assistance, and how to recognize and avoid possible co-factors, which may multiply the risk for severe anaphylaxis [50].

In the last few years, Internet and social media have become highly accessible information sources for health-related queries [97]. The few studies that have focused on the impact of these technologies in patients with anaphylaxis tend to describe the beneficial effects, as in other allergic diseases. The use of Internet, social media, and mobile applications may play a role in future approaches to education in anaphylaxis [98–100].

## Anaphylaxis in special populations

Various groups of patients present particularities that affect how anaphylaxis should be managed in the ED. These particularities should also be taken into account when assessing the risk of anaphylaxis and establishing preventive measures.

Infants may not be able to describe their anaphylaxis symptoms properly, and some signs may be difficult to interpret (irritability, crying, somnolence, etc.), thus delaying diagnosis and treatment. The clinical criteria for diagnosis of anaphylaxis in the ED have not been specifically validated for use in this age group. The differential diagnosis of anaphylaxis in infants must also include congenital abnormalities, aspiration of a foreign body, or food protein-induced enterocolitis syndrome, which seldom occur later in life [101].

Food allergy is the most common cause of anaphylaxis in childhood and has become a common health issue in schools [102]. Around 20% of cases of anaphylaxis may occur in this setting [103, 104]. Nevertheless, many schools are insufficiently prepared to manage anaphylaxis [105], with limited availability of emergency action plans, epinephrine, and trained school staff, thus delaying diagnosis and transfer of patients to the ED, where management can be hampered by the lack of reliable information. In order to improve the management of anaphylaxis in schools, individualized measures should include collaboration between parents, school personnel, and allergists or paediatricians [106].

Teenagers are at greater risk for anaphylaxis owing to the intrinsic characteristics of this age group [98, 107, 108]. Adolescents tend to have higher risk behaviour and thus minimize the consequences of transgressions, thus potentially leading them to disregard triggers of anaphylaxis. They also try to hide their allergy problems from others, avoid EAIs, and seek medical care only at late stages of the reaction. These factors may delay the recognition of an episode of anaphylaxis. Management of anaphylaxis in teenagers presenting at the ED may be hampered by misinformation (e.g., lessening of symptoms, hiding triggers) [109, 110]. The first experiences with alcohol may also act as a co-factor of severity [93].

Old age does not seem to increase the risk of anaphylaxis [111]. However, it has been associated with a higher risk of death, possibly as a consequence of comorbidities, polypharmacy, higher risk of hospitalization, and changes in the immune system, which lead to a pro-inflammatory state [112]. In elderly patients with anaphylaxis managed in the ED, age or even a history of cardiovascular disease is not an absolute contraindication for the administration of epinephrine. Nevertheless, the potential advantages and disadvantages must be carefully considered [76].

The prevalence of anaphylaxis, especially idiopathic anaphylaxis, is higher in patients with mastocytosis than in the general population [113]. NSAIDs and hymenoptera venom hypersensitivity are also frequent among these patients. Evaluation of patients with mastocytosis in the ED must take into consideration that anaphylaxis is particularly severe in these cases, with cardiovascular symptoms being very common. In many cases, no eliciting trigger can be identified [114, 115]. Patients with underlying mastocytosis should always be prescribed at least one EAI [116].

## Conclusions

In summary, anaphylaxis may not be as uncommon as previously thought, and epidemiologic publications are prone to discrepancies owing to the different methodologies, target populations, and settings.

Anaphylaxis is not always well recognized, especially if hypotension is the only sign. This multisystemic disease may present as very different combinations of symptoms, and apparently mild signs may unpredictably progress to fatal anaphylactic shock. A rapid diagnosis leads to optimal management. Fast intervention is critical. Estimation of circulatory, respiratory, and mental status and removal of the possible cause should be followed by administration of intramuscular epinephrine, which is the treatment of choice, with no absolute contraindications. Moreover, the risk–benefit ratio should always be assessed in patients with cardiovascular disease. Antihistamines and corticosteroids are second-choice medications. An EAI should always be prescribed after a suspected episode of anaphylaxis.

Etiologic factors suspected in the ED often differ from the real cause. Nonetheless, since the ED is not the appropriate place to study the cause of the anaphylaxis, a meticulous allergy workup should be offered. Special attention should be given to co-factors, as these may easily confound the cause of anaphylaxis.

Finally, anaphylaxis is a complex disease that should be well recognized and handled by any physician. We stress the need for increased awareness of anaphylaxis among health professionals, who should receive appropriate training to diagnose and manage it.

## Abbreviations

ACE: angiotensin-converting enzyme
BP: blood pressure
EAI: epinephrine auto-injector
ED: emergency department
ICD: International Classification of Diseases
NSAID: nonsteroidal anti-inflammatory drug
PEF: peak expiratory flow
